# A systematic review of studies on resilience and risk and protective factors for health among refugee children in Nordic countries

**DOI:** 10.1007/s00787-022-01975-y

**Published:** 2022-04-20

**Authors:** Erica Mattelin, Kristina Paidar, Natalie Söderlind, Frida Fröberg, Laura Korhonen

**Affiliations:** 1 Barnafrid and Department of Biomedical and Clinical Sciences, Linköping University, Linköping, Sweden; 2 Center for Social and Affective Neuroscience and Department of Biomedical and Clinical Sciences, Linköping University, Linköping, Sweden; 3 Department of Child and Adolescent Psychiatry, Region Halland, Kungsbacka, Sweden; 4 Department of Child and Adolescent Psychiatry and Department of Biomedical and Clinical Sciences, Linköping University, Linköping, Sweden

**Keywords:** Refugee, Child, Health, Risk and protective factors, Resilience, Systematic review, Nordic countries

## Abstract

The Nordic welfare model is often used as an example for the promotion of health and wellbeing, even in vulnerable groups of children, such as refugees. Nonetheless, there are no published reviews on resilience and/or risk and protective factors for physical and mental health among refugee children living in Nordic countries. In this systematic review, we identified 5181 studies on the topic, screened titles, and abstracts, viewed 632, and finally included 26 studies. These studies described 18 samples with a total of 34,080 individuals ranging in ages 0–18 years. Overall, the studies were of good quality. Nearly all studies assessed adversity. Six studies reported physical health outcomes and all studies mental health outcomes, most often post-traumatic stress disorder and anxiety. None explicitly studied resilience. While we found that age and sex are the most frequently studied  risk- and protective factors, findings are inconclusive, since the direction of the associations was different in the different studies. This systematic review indicates that there is still a need for well-designed and -powered studies using clear definitions of key study concepts to examine health outcomes and resilience among refugee children in Nordic countries.

**Supplementary Information:**

The online version contains supplementary material available at 10.1007/s00787-022-01975-y.

## Background

According to the United Nations, over 80 million people are displaced, and about half of them are minors. The population is heterogeneous and includes asylum-seekers and refugees fleeing with or without their families. Reasons to flee include acute threats, such as war and conflicts, violation of human rights, famine, and political instability [71].

The migrant flows in the Nordic Region have changed dramatically during the past decades from primarily intra-Nordic work-related migration to an influx of asylum-seekers from different parts of the world [36]. During the refugee crisis in 2015, Nordic countries, especially Sweden, received many asylum applications per capita compared to many other European countries. Among the Nordic countries, Sweden used to stand out as a country with an inclusive immigration policy, but this has been restricted in the past years [69]. Children make up 25–35% of refugees and come to the Nordic region as quota refugees or by other means [63]. From 2006 to 2018 asylum was granted in Nordic countries to approximately 400,000 refugees, mainly coming from Afghanistan, Iran, Iraq, Somalia, other African countries, and Syria [63]. Most of the refugees have settled in Sweden [63].

## Exposure to adversity and negative health-related consequences

Forced migration is characterized by its complexity. It often involves moves between different countries, camps, and accommodations. Experiences range from relatively straightforward routes to travels between countries, longer stays in camps, and sometimes being dependent on smugglers. Refugee children may experience many types of adversities at one or more stages of the migration process: in their countries of origin, on the move, and in the country of resettlement [34]. Adversities include violence and other factors in the child’s environment that can undermine a sense of safety, stability, and bonding.

Exposure to negative experiences is common among refugee children. Studies indicate high rates of child labor [24], financial problems [31], poor access to nutrition [17], and a high prevalence of physical (9–65%) and sexual (5–20%) violence among migrant children in general [34]. However, these numbers should be interpreted with caution, since high-quality data is lacking [34].

The effects of exposure to adverse events are well documented [19, 21], and include poor mental health, physical health problems [21, 26], and later exposure to violence [77]. A recent meta-analysis estimated a prevalence of 22.71% for post-traumatic stress disorder (PTSD), 13.81% for depression, and 15.77% for anxiety disorders among refugee and asylum-seeking children worldwide in 2003–2018 [11]. In comparison with non-refugee children, both in the countries of origin and resettlement, these numbers stand out as high [11]. A recent systematic review on risk and protective factors for mental health concluded that no or low exposure to violence, stable settlement, and social support are associated with better outcomes in the country of settlement [18]. None of these systematic reviews have focused on Nordic countries.

Apart from mental health problems, refugee children have considerable physical health needs on arrival in reception countries. A recent systematic review [5] found high prevalence rates of hematological conditions, such as anemia and different infections, such as chronic hepatitis B and latent tuberculosis. In addition, vitamin D deficiency and other nutrition-related problems, as well as oral health problems, are common. For example, the European Association of Paediatrics and the European Commission have developed guidelines for providing medical care to refugee children entering European countries [16, 61]. According to these guidelines, health assessments should be based on individual needs depending on conditions before, under, and after displacement.

## Resilience

As of today, migrant studies have focused on adversity and mental health problems [8], although it is known that some individuals can adapt and integrate into the new environment despite significant adversities [56, 57]. This ability to prosper after adversities, often referred to as resilience, has received increasing empirical attention [41–43]. However, there is no consensus on its definition [67]. The concept has evolved from an early emphasis on a positive outcome in the individual, such as the absence of mental health problems, to a contemporary conceptualization of resilience as “the capacity of a system to adapt successfully to disturbances that threaten the viability, function, or development of the system” [41, 42]. Still, exposure to adversity and an adaptation or positive outcome are the core components of resilience [67].

Resilience-related outcomes studied among child refugees include self-efficacy, self-esteem, and quality of life [40]. Furthermore, these outcomes are associated with young age, maintenance of cultural identity, social support, sense of belonging, safety, and innovative social care service [40]. This emphasizes the importance of focusing on the child’s physical and social ecology when trying to unfold resilience and ways to facilitate health and wellbeing despite adverse experiences [72]. At the same time, more stringent studies on resilience-related factors, i.e., stable trait-like characteristics or predispositions, resilience process, and resilient outcomes, are warranted to understand the complex and dynamic interplay between different factors and circumstances upon experiences with adversity [35].

## Nordic welfare societies, refugee health, and resilience

The countries in the Nordic region have similar societal structures and cultural and historical backgrounds. The Nordic welfare model, a unique combination of market economy, social benefits, and high quality of life [70], is often used as an example of promoting health and redistributing wealth. All Nordic countries have health reception policies that consider both physical and mental health among arriving refugees [63]. Regarding national policies, refugee children have the same access to welfare measures, including access to health care, education, and social services [22, 63]. However, Denmark, compared to other Nordic countries, has more restrictive policies related to financial support, family reunification, and the possibility of acquiring citizenship according to the EU Migration Policy Index [9, 63]. In addition, asylum seekers in Denmark are excluded from entitlements to upper secondary school, and there is no national legislation to ensure refugee children equal health care services compared to resident children [63].

A recent report has pointed out several inequalities between refugees and the native-born population across the Nordic region in education achievement and health, with unaccompanied refugee minors having greater inequalities in comparison to accompanied minors [63]. In line with this, the general aim of this systematic review is to elucidate knowledge about resilience and risk and protective factors for mental and physical health among refugee children living in Nordic countries. This knowledge could be used to tailor measures and investments in efforts to tackle health-related inequalities as well as to promote healthy development and resilience in refugee children.

## Methods

### Design

Reporting follows the preferred reporting items systematic reviews and meta-analysis (PRISMA) guidelines [46]. The protocol was submitted for registration in Prospero on September 30, 2019, and registered on July 10, 2020.

### Eligibility criteria

The study population was child refugees (i.e., asylum seekers, refugees, family migrants, quota refugees, undocumented; mean age of the study population was 18 years or younger) residing in a Nordic country (Denmark, Finland, Norway, Sweden, and Iceland). The outcomes of interest were quantitative measures related to physical and mental health (e.g., symptoms and diagnoses), health-related outcomes (e.g., adaptation, recovery, and adjustment), risk and protective factors (e.g., age, sex, and exposure to adversity), and resilience (e.g., sense of coherence, post-traumatic growth, and self-management). Risk and protective factors were defined based on the following criteria: a) there was an association between an exposure and outcome, b) risk and protective factor occurred before the outcome, c) a clear reason was stated for a factor to be considered a risk or protective factor.

Qualitative studies, case reports, studies with very small participant numbers (*n *< 10), discussion papers, commentaries, editorials, letters, book chapters, conference papers, books, doctoral theses, and dissertations were excluded. Studies had to be published in English, Swedish, Norwegian, Danish, Finnish, or Icelandic language journals.

### Literature search

The data search was conducted in September 2019 and December 2021. The used databases were PubMed, Scopus, PsychINFO, Web of Science, CINAHL ERIC, Libris, and Cochrane. The search strategy is presented in Appendix 1. In total, 5181 articles were identified via database search. The initial search was supplemented by a manual search of reference lists of included reports, relevant books, and reviews on the subject and using forward and backward citations. This yielded one additional report.

### Study selection

**Fig. 1:**
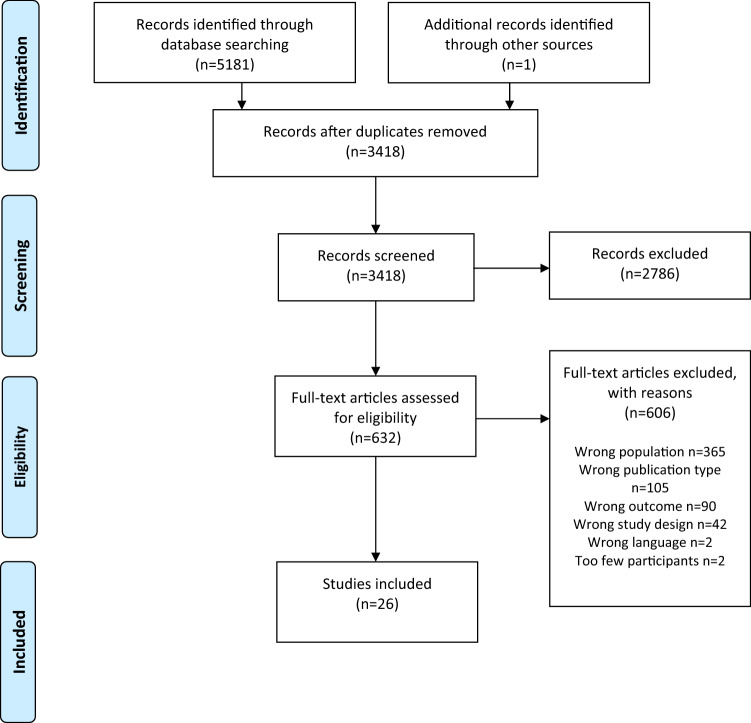
PRISMA 2009 Flow Diagram

After removing duplicates, 3418 records were screened at the title and abstract level by EM and KP independently from each other. At this stage, 2786 studies were excluded, and the remaining 632 studies were selected for further reading in full text (EM and KP). Subsequently, 606 articles were excluded, because they did not fulfill the inclusion criteria: (1) migrant status (i.e., asylum seekers, refugees, family migrants, quota refugees, undocumented) clearly defined; (2) study sample consists of migrants residing in a Nordic country; (3) study population consists of individuals with mean age max 18 years; (4) study reports outcome data on health or health-related factors; and (5) study reports data on risk or protective factors or resilience. All conflicts in inclusion/exclusion were resolved through discussion between EM, KP, NS, FF, and LK. Causes for exclusion were documented for each study. LK and FF controlled all included and excluded studies. An overview of the inclusion process and reasons for exclusion is shown in the flow chart (Fig. 1). A list of excluded publications can be requested from the corresponding author.

### Study quality and risk for bias

The quality assessment was performed by EM and KP independent from each other using the Standard Quality Assessment Criteria for Evaluating Primary Research Papers “QualSyst”. The checklist consists of 14 items that are scored as 0 (No), 1 (Partial), 2 (Yes), or not available (n/a) [38]. (1) Is the question/objective sufficiently described?; (2) Is the study design evident and appropriate?; (3) Is the method of subject/comparison group selection or source of information/input variables described and appropriate?; (4) Are the subject (and comparison group, if applicable) characteristics sufficiently described?; (5) If the interventional and random allocation was possible, was it described?; (6) If the interventional and blinding of investigators was possible, was it reported?; (7) If the interventional and blinding of subjects was possible, was it reported?; (8) Is/are the outcome and (if applicable) exposure measures(s) well defined and robust to measurement/misclassification bias?, Are the means of assessment reported?; (9) Is the sample size appropriate?; (10) Are the analytic methods described/justified and appropriate?; (11) Is some estimate of variance reported for the main results?; (12) Are the results controlled for confounding?; (13) Are the results reported in sufficient detail?; (14) Do the conclusions support the results?.

A summary score (range 0–1) was calculated for each study by summing the total score obtained across relevant items and dividing the total possible score (i.e., 28—(number of “n/a” × 2)). Studies were categorized based on the summary score as follows: strong study (score of > 0.80), good study (0.71–0.79), adequate study (0.50–0.70), and limited study (< 0.50). The inter-rater reliability, based on 10% of randomly selected studies, was ICC = 0.92 for the total score, indicating excellent inter-rater reliability [39]. Differences between ratings  were discussed to reach an agreement. Overall, the studies were of good quality, with an average summary score of 0.83 (range 0.55–1.0). 24 out of 26 (85%) included studies scored 0.71 and above, indicating a good or strong study.

### Ethical considerations

13 out of 26 (50%) studies did not report ethical approval. This could be explained by the fact that no ethical approval is needed for register studies in Denmark (*n* = 2) and that several studies from Sweden (*n* = 8) were conducted before the legislation on ethical approval was enacted in 2004. There is a surprisingly low drop-out rate and data loss, which is sparsely discussed in the studies.

### Data extraction and data items

Data extraction was performed by EM, KP, and NS with subsequent review by LK and FF. From each selected study, the following information on the study type and population was extracted: study origin, study design, data collection year(s), sample size, age (mean age and range), % females, response rate, migrant status, country/countries of origin of the migrants, and ethical approval. For the analysis of health-related outcomes and risk and protective factors, the following information was extracted: physical and mental health as well as other health-related outcomes, risk and protective factors, resilience outcome, used instrument, and type of informant. In addition, data on exposures to adversity, stressful life events, and/or violence was extracted.

## Results

Table 1 summarizes the included 26 studies that described 18 samples and were published between 1991 and 2021. The studies covered 34,080 participants and represented all Nordic countries except Iceland: Sweden (*n* = 13), Denmark (*n* = 9), Norway (n = 3), Finland (n = 1). The time of data collection ranged from directly at arrival to 9 years (follow-up) and was done between 1986 and 2019. 17 studies had a cross-sectional design, and the rest were follow-up and cohort studies. The largest sample size was in a Danish register study (*n* = 16,464). 12 studies (46.2%) had sample sizes below 100 (range *n* = 29–99).

**Table 1:** Articles included in the systematic review

	Authors and publication year	Title	Data collection year(s)	Study design	Study country	Sample size (*n*), % females	Study population	Migrant country of origin	Study participant age-range, mean age (M)	Response rate (%)	Measurements	Outcomes within scope of this review	Quality assessment score	Ethical app-roval
1	Abdalla and Elklit [1]	A nationwide screening of refugee children from Kosovo	1999	Cross sectional study	Denmark	1224, 48%. Sex was unknown for 8% of the participants	Refugee children	Kosovo	0–18 years, *M* = 8.2 years	89.2%	Medical examinations, screening with a non-validated trauma- and symptom questionnaire	Problems with sleep, eating, vision, hearing, enuresis, encopresis, urinary infection, headache, toothache, motor issues, anxiety, nervousness, PTSD, depression, aggression, psychosomatic symptoms, regressive traits, behavioral problems	55	No
2	Almqvist and Brandell-Forsberg [2]	Refugee children in Sweden: post-traumatic stress disorder in Iranian preschool children exposed to organized violence	1987–1991	Prospective longitudinal study	Sweden	50,28%	Refugee children	Iran	3–8 years, *M* = 5.8 years	96% (reported on family level)	Semi-structured interviews with parents, child assessments	PTSD	81	No
3	Almqvist and Broberg [3]	Mental health and social adjustment in young refugee children 3.5 years after their arrival in Sweden	2.5years after 1988–1989	Prospective longitudinal study	Sweden	39, 26%	Refugee children	Iran	6–10 years, *M* = 8.3 years	78%	Semi-structured interviews with parents and children	Children’s mental health including PTSD	86	No
4	Angel et al. [4]	Effects of war and organized violence on children: a study of Bosnian refugees in Sweden	1994–1995	Cross sectional study	Sweden	99, NR	Refugee children	Bosnia-Hercego-vina	6–16 years, *M* = 11.3 years	91% (reported on family level)	Semi-structured interviews, short version of a questionnaire developed by Cederblad and Höök	PTSD-related symptoms, anxiety, sad mood, sleep disturbance, poor appetite, separation anxiety, concentration problems, restlessness, withdrawn, recurrent abdominal pain, enuresis, aggressive, defiance, tics, headaches	75	No
5	Back Nielsen et al. [6]	Risk of childhood psychiatric disorders in children of refugee parents with post-traumatic stress disorder: a nationwide, register-based, cohort study	1995–2015	Retro-spective cohort study	Denmark	51,793*, 48.6% *16,464 first genera-tion	Refugee children and bio-logical children of refugees	Central Asia, Central Europe, Sub-Saharan Africa, Eastern Europe, Middle East, other	0–18 years, *M* = NR	NA	Nationwide Danish population-based registers	Substance use disorders, psychotic and affective disorders, nervous disorders, disorders of psychological development, behavioral and emotional disorders	100	No
6	Berg et al. [7]	Under-utilisation of psychiatric care among refugee adolescents in Stockholm	2011–2017	Cross-sectional	Sweden	93,537 5003 asylum seeking children (48.8%) and 2635 children that applied for family reunify-cation (52.2%)	Asylum-seeking children and children that had sought family re-unification	Europe, North America, East Asia, Iraq, Syria, Iran, Asia, Africa, South America, Somalia, Eritrea, Afghanistan, South Asia, Vietnam	11–18 *M* = NR	NA	Main diagnosis according to ICD-10	First visit to a child psychiatric unit and main diagnosis according to ICD-10	91	Yes
7	Eiset et al. [14]	The health status of newly arrived asylum-seeking minors in Denmark: a nationwide register-based study	2011–2015	Cross-sectional	Denmark	7210, 43%	Asylum seeking minors	Syria, Russia, Stateless, Other	0–17 *M* = NR	NA	Medical examinations, screening with a non-validated trauma- and symptom questionnaire	Anxiety, depression, need of intervention or clinical support	91	Not needed
8	Ekblad [15]	Psychosocial adaptation of children while housed in a Swedish refugee camp: Aftermath of the collapse of Yugoslavia	1992	Cross sectional study	Sweden	66, 50%	Refugee children in refugee camps in Sweden	Former Yugoslavia	5–15 years, *M* = NR	NR	Semi-structured interviews	Fear, nightmares, regression in development, aggressiveness, repetition of trauma in play, depression, somatic symptoms	63	No
9	Gusic et al. [23]	Dissociative experiences and trauma exposure among newly arrived and settled young war refugees	NR	Cross sectional study	Sweden	77, In two different groups: 1: *n* = 42 (38%) 2: *n* = 35(31%)	Refugee adolescents and settled ado-lescents with experience of war	Afghanistan, Iraq, Lebanon, North Africa, Somalia, Syria, Other Middle East countries	Group 1: 13–19 years, *M* = 16.1 years Group 2: 11-18 years, *M* = 14.8 years	NR	A-DES, CRIES	PTSD and dissociative symptoms	77	Yes
10	Hjern et al. [28]	Persecution and behavior: A report on refugee children from Chile	1986–1987	Cross sectional study	Sweden	50, 48%	Refugee children	Chile	2–15 years, *M* = 5.9 years (0–5 months before the interview)	90.9%	Semi-structured interviews with children and parents	Sleep disturbance, dependency, anxiety, depressed mood, concentration difficulties, withdrawal, hyperactivity, defiance, aggressiveness, recurrent abdominal pain, headache, poor appetite, enuresis	68	No
11	Hjern et al. [29]	Political violence, family stress and mental health of refugee children in exile	1986–1987	Cross-sectional study	Sweden	63, NR	Refugee children	Chile, Iran, Lebanon, Turkey	2–15 years, *M* = 5.9 years on arrival to Sweden	81.8%	Clinical interviews with parents, written interviews with teacher	Sleep disturbance, dependency, anxiety, depressed mood, concentration difficulties, withdrawal, hyperactivity, defiance, aggressiveness, recurrent abdominal pain, headache, poor appetite, enuresis	73	No
12	Hjern and Angel [27]	Organized violence and mental health of refugee children in exile: a 6-year follow-up	6–7 years after 1986–1987	Prospective cohort study	Sweden	49, NR	Refugee children	Chile, Iran, Lebanon, Turkey	8–20 years, *M* = 12.8 years	77.8%	Clinical interviews with parents and children including a non-validated questionnaire^1^, child behaviors questionnaire for teachers developed by Rutter	Sleep disturbance, dependency, anxiety, depressed mood, concentration difficulties, withdrawal, hyperactivity, defiance, aggressiveness, recurrent abdominal pain, headache, poor appetite, enuresis, post-traumatic stress	77	Yes
13	Jensen et al. [32]	Stressful life experiences and mental health problems among unaccompanied asylum-seeking children	2010–2012	Cross-sectional study	Norway	93, 19% or 16%, different numbers are reported in the article)	Unaccompa-nied refugee children	Afghanistan, Eritrea, Ethiopia, Iraq, Somalia, Sri Lanka, Other countries (Asia, Africa, Europe)	10–16 years, *M* = 13.8 years	NR	CPSS, HSCL-37	PTSD, externalizing and internalizing symptoms, anxiety and depressive symptoms	95	Yes
14	Jensen et al. [33]	Development of mental health problems—a follow- up study of unaccompa-nied refugee minors	2012–2013	Prospective cohort study	Norway	75, 17%	Unaccompa-nied refugee children	Afghanistan, Eritrea, Somalia, Sri Lanka, others	13.5–20.7 years, M = 16.5 years	NR	CPSS, HSCL-37	PTSD, externalizing and internalizing symptoms, anxiety and depressive symptoms	95	Yes
15	Mont-gomery and Foldspang [52]	Traumatic experience and sleep disturbance in refugee children from the Middle East	1992–1993	Cross-sectional study	Denmark	311, 49%	Refugee children	Iran, Iraq, Lebanon, Syria, Stateless Palestinians, Turkey	3–15 years, *M* = 7.5 years	90.4%	Structured interview	Sleep disturbances	70	Yes
16	Montgo-mery and Foldspang [51]	Seeking asylum in Denmark: refugee children’s mental health and exposure to violence	1992–1997	Prospective cohort study	Denmark	311, 49%	Refugee children	Middle East (countries/ethnicities defined as Iraq, Stateless Palestinians and Kurds)	3–15 years, *M* = 7.5 years	NR	Structured interview and register data	Anxiety, sad or miserable appearance, sleep disturbances	75	Yes
17	Montgomery [48]	Long-term effects of organized violence on young Middle Eastern refugees’ mental health	2001	Prospective cohort study	Denmark	131, 58%	Refugee children	Iran, Iraq, Lebanon, Stateless Pales- tinians, Syria	11–23 years, *M* = 15.3 years	72%	YASR, YSR, structured interviews	Internalizing and externalizing behaviors	82	Yes
18	Montgomery [49]	Trauma and resilience in young refugees: A 9-year follow-up study	1992–2001	Prospective cohort study	Denmark	131, 58%	Refugee children	Iran, Iraq, Stateless Palestinians	11–23 years, *M* = 15.3 years	72%	CBCL, YABC, YASR, YSR, structured and semi-structured interviews	Internalizing and externalizing behavior's, anxiety, depressive symptoms, sleep disturbance	86	Yes
19	Montgomery and Foldspang [50]	Discrimination, mental problems and social adaption in young refugees	1992–2001	Prospective cohort study	Denmark	131, 58%	Refugee children	Iran, Iraq, Lebanon, Stateless Palesti-nians, Syria	11–23 years, *M* = 15.3 years	72%	YASR, YSR, structured interviews	Externalizing and internalizing behavior	9O	Yes
20	Nielsen et al. [53]	Mental health among children seeking asylum in Denmark—the effect of length of stay and number of relocations: a cross-sectional study	2006	Cross sectional study	Denmark	246, 42%	Asylum seeking children	Afghanistan, Armenia, *Azerba-jdzjan*, Iran, Kazakhstan, Libya, Lithuania, Pakistan, Russia, Somalia, Sri Lanka, The State of Palestine, Syria, Ukraine, Former Yugoslavia	4–16 years, *M* = 9.6 years	95%	SDQ	Emotional symptoms, hyperactivity, conduct problems	95	No
21	Salari et al. [58]	Screening for PTSD symptoms in unaccompanied refugee minors: a test of the CRIES-8 questionnaire in routine care	2015–2016	Cross-sectional study	Sweden	208, 2.4%	Asylum-seeking unaccompanied children	Afghanistan, Eritrea, Ethiopia, Iran, Iraq, Lebanon, Libya, Syria, Pakistan, Somalia	9–18 years, *M* = 15.4 years	Unclear	CRIES-8	PTSD	95	Yes
22	Sarkadi et al. [59]	Is the Refugee Health Screener a Useful Tool when Screening 14-to 18-Year-Old Refugee Adolescents for Emotional Distress?	2017	Cross-sectional	Sweden	29, 24.1%	Refugee children	Afghanistan, India, Iraq, Iran, Sri Lanka, Syria, Venezuela	14–18 *M* = 16.55years	100%	Refugee Health Screener (RHS) and CRIES-8	Emotional distress and PTSD	73	No
23	Solberg et al. [66]	Children at risk: A nation-wide, cross-sectional study examining post-traumatic stress symptoms in refugee minors from Syria, Iraq and Afghanistan resettled in Sweden between 2014 and 2018	2018	Cross sectional study	Sweden	1129, 46.9%	Refugee minors	Syria, Iraq and Afghanistan	16–18 *M* = NR	22.3%	Questionnaire and register variables including CRIES-8 and demographic information	PTSD	100	Yes
24	Solberg et al. [65]	Health-related quality of life in refugee minors from Syria, Iraq and Afghanistan resettled in Sweden: a nation-wide, cross-sectional study	2018	Cross sectional study	Sweden	2559, 45%	Refugee minors	Syria, Iraq and Afghanistan	12–18 *M* = NR	26%	KIDSCREEN-27 and socio-demographic information	Health-related quality of life	100	Yes
25	Sourander [66]	Behavior problems and traumatic events of unaccompanied refugee minors	1994–1995	Cross sectional study	Finland	46, 26%	Unaccompanied children	Angola, Burma, Ethiopia, Iraq, Nigeria, Somalia, Thailand, Vietnam, Zaire	6–17 years, *M* = 14.1 years	NR	CBCL, clinical records, legal document, documents on police, interviews on arrival, staff members´ and children´s interviews	Behavior problems	77	No
26	Vervliet et al. [76]	The mental health of unaccompanied refugee minors on arrival in the host country	2009–2011	Cross sectional study	Norway and Belgium. We only present data on participants from Norway	204, 0%	Unaccompanied refugee minors	Afghanistan, Algeria, Iran, Somalia, Palestine, West Sahara	NR, *M* = 16.2 years	94%	HSCL-37A, PTSS16	Anxiety, depression, internalizing problems, PTSD	90	No

Scales and instruments in table are: *A*-*DES* adolescent dissociative experiences Scale, *CBCL* child behavior check list, *CPSS* child PTSD symptom scale, *CRIES* children´s revised impact of event scale, *HSCL-37/HSCL-37A* Hopkins symptom checklist-37, *PTSD* post-traumatic stress disorder, *PTSS-16* post traumatic symptom *scale*-16, *SDQ* strengths and difficulties questionnaire *YABC* young adult behavior checklist, *YASR* young adult self-report, *YSR* youth self-report. Number are: *n* (count), % (percentage) and *NR* not reported

The children (*n* = 34,080) were between 0 and 18 years. Five studies (19.2%) focused on unaccompanied minors and the remaining 21 on refugees and/or asylum-seeking children. In six studies (23.1%), the migrant country of origin was limited to one (Bosnia-Herzegovina, Chile, Iran, Kosovo, or former Yugoslavia), whereas the other studies included participants from several countries and continents (Asia, Africa, Europe, South America).

In the following, we describe the reported health, resilience, and other outcomes (Table 1) from the included 26 studies. Analysis of risk and protective factors for mental and physical health including exposure to adversity, stressful life events, and/or violence are shown in Table 2 and Table 3. For this purpose, risk and protective factors have been classified based on the content in the individual, family as well as community, and society level factors following the ecological system theory and other systematic reviews into the topic [12, 18,31,55].

**Table 2:** Exposure to stressful life events, adversity and/or violence

	Authors and publication year	Title	Reported exposures to stressful life events, adversity and/or violence
1	Abdalla and Elklit [1]	A nationwide screening of refugee children from Kosovo	Witness to violence, exposed to violence, exercised violence, participated in acts of war, physical damage, extreme poverty, starvation, torture, separation and loss
2	Almqvist and Brandell-Forsberg [2]	Refugee children in Sweden: post-traumatic stress disorder in Iranian preschool children exposed to organized violence	Exposure to political persecution and war experiences
3	Almqvist and Broberg [3]	Mental health and social adjustment in young refugee children 3.5 years after their arrival in Sweden	Children’s exposure to traumatic stress, parental exposure to traumatic stress, children’s exposure to bullying and harassment
4	Angel et al. [4]	Effects of war and organized violence on children: a study of Bosnian refugees in Sweden	Experiences of war and persecution
5	Back Nielsen et al. [6]	Risk of childhood psychiatric disorders in children of refugee parents with post-traumatic stress disorder: a nationwide, register-based, cohort study	N/A
6	Berg et al. [7]	Underutilisation of psychiatric care among refugee adolescents in Stockholm	N/A
7	Eiset et al. [14]	The health status of newly arrived asylum-seeking minors in Denmark: a nationwide register-based study	Separations, loss, poverty or starvation, violence
8	Ekblad [15]	Psychosocial adaptation of children while housed in a Swedish refugee camp: Aftermath of the collapse of Yugoslavia	Separation/losses, exposure to violence, e.g., seeing police and military vehicles
9	Gusic et al. [23]	Dissociative experiences and trauma exposure among newly arrived and settled young war refugees	War/refugee and general trauma
10	Hjern et al. [28]	Persecution and behavior: a report on refugee children from Chile	Experiences related to political persecution (e.g., witnessed assaults and arrests or exposure to violence)
11	Hjern et al. [29]	Political violence, family stress and mental health of refugee children in exile	Organized violence (e.g., witness to violence) and separations
12	Hjern and Angel [27]	Organized violence and mental health of refugee children in exile: a 6-year follow-up	Organized violence and recent family stress (e.g., death of parent)
13	Jensen et al. [32]	Stressful life experiences and mental health problems among unaccompanied asylum-seeking children	Separation from family, physical or sexual violence, and war or armed conflict
14	Jensen et al. [33]	Development of mental health problems—a follow-up study of unaccompanied refugee minors	Separation from family, physical or sexual violence, and war or armed conflict
15	Montgomery and Foldspang [52]	Traumatic experience and sleep disturbance in refugee children from the Middle East	Exposure to war and other organized violence
16	Montgomery and Foldspang [51]	Seeking asylum in Denmark: refugee children’s mental health and exposure to violence	Lived under conditions of war, witnessed violence, parents detained or tortured, own detainment or parent death or disappearance
17	Montgomery [48]	Long-term effects of organized violence on young Middle Eastern refugees’ mental health	Experiences before arrival in Denmark (e.g., lived under war conditions, lived in a refugee camp), experiences after arrival in Denmark (been attacked, witnessed attack), discrimination in Denmark (e.g., teased, derogatory remarks)
18	Montgomery [49]	Trauma and resilience in young refugees: a 9 year follow-up study	War related life conditions, witnessing violent acts, loss and separation, direct exposure to violence, family exposure
19	Montgomery and Foldspang [52]	Discrimination, mental problems and social adaption in young refugees	Discrimination
20	Nielsen et al. [53]	Mental health among children seeking asylum in Denmark—the effect of length of stay and number of relocations: a cross-sectional study	N/A
21	Salari et al. [58]	Screening for PTSD symptoms in unaccompanied refugee minors: a test of the CRIES-8 questionnaire in routine care	N/A
22	Sarkadi et al. [59]	Is the Refugee Health Screener a Useful Tool when Screening 14–18 Year-Old Refugee Adolescents for Emotional Distress?	N/A
23	Solberg et al. [66]	Children at risk: a nation-wide, cross-sectional study examining post-traumatic stress symptoms in refugee minors from Syria, Iraq and Afghanistan resettled in Sweden between 2014 and 2018	N/A
24	Solberg et al. [65]	Health-related quality of life in refugee minors from Syria, Iraq and Afghanistan resettled in Sweden: a nation-wide, cross-sectional study	N/A
25	Sourander [66]	Behavior problems and traumatic events of unaccompanied refugee minors	Death or disappearance of parent, eyewitness to violence, personal experience of violence, lived in refugee camp and persecution
26	Vervliet et al. [76]	The mental health of unaccompanied refugee minors on arrival in the host country	Separation from family, physical or sexual violence, and war or armed conflict

*N/A* not applicable

### Outcomes

#### Health-related outcomes

As shown in Table 1, most of the data on health-related outcomes focused on physical and mental health and were collected from the parents. Only one study reported on health care utilization and one study on health-related quality of life. Several studies (38.5%) did not use any standardized instruments, and only a few studies have used register data.

Six (23.1%) studies reported data on physical health outcomes, such as poor appetite, recurrent abdominal pain, enuresis, parasitic infections, headache, poor appetite, or consolation eating [1,4, 14, 15, 27–29]. These studies are mainly Swedish, focusing on refugees who arrived from Balkan, the Middle East, and Chile at the end of the 1980s.

All studies reported mental health outcomes, most commonly PTSD, anxiety, and depression. Prevalence of depression ranged from 2 to 45%, PTSD from 2.9 to 76.4%, and anxiety from 5-69%. Other commonly reported mental health outcomes were sleep disorders (prevalence range 5.4-75%) and behavioral problems (prevalence range 2.2-25%). Almqvist and Broberg found that 26% of the children in their study had good emotional well-being at the first timepoint and 38% at the second 2.5 years later [3]. In another study by Hjern, Angel and Jeppson, more than half of the children in the sample had no mental health problem at baseline (54%) or 17–19 month follow-up (56%), and the number was even higher after 6–7 years (78%) [27] [29]. It is, however, worth noticing that there might be an erratum in prevalence, since different figures are given in articles [29] and [27].

One Swedish study [7] investigated psychiatric care utilization and showed that the use of psychiatric care among asylum seekers was higher than in those who settled in family reunification. Another study found that refugee minors in Sweden had a higher health-related quality of life than a European comparison group on two dimensions, relation to parents and autonomy and school environment. However, the opposite was seen for psychological wellbeing, and social support [65].

### Resilience

None of the identified studies explicitly investigated resilience and/or related factors following any of the current definitions or conceptualizations.

## The individual risk or protective factors

### Age, sex, and socioeconomic status

As shown in Table 3, 15 studies examined the association between age (at the time of the study) and health outcomes among refugee children with mixed results. Many studies did not show any associations between age and mental health [3, 4, 23, 29, 32, 58, 64, 76]. Other studies showed mixed results. For example, a Danish study by Abdalla and Elklit of refugee children from Kosovo [1] found that older age was associated with more symptoms of PTSD, anxiety, nervousness, and headache, but with fewer symptoms of enuresis and problems with eating, compared to a younger age. Similarly, a Danish study by Montgomery and Foldspang [50] found that older age was associated with a higher likelihood of developing internalizing symptoms. In contrast, in a Finnish study [66] younger age was associated with a higher risk of behavioral problems (OR 4.1; 95% CI 1.1–15.0), similar to a Danish study finding that higher age at the follow-up to the baseline assessment was negatively associated with externalizing symptoms  [48].

16 studies investigated the association between sex and health outcomes. Most of the articles included in this review report no association between sex and health among refugee children [1] [4] [27] [33] [66] [76] [23] [6]. In the few studies that found an association, the results were inconclusive. Some stated that the female sex was negatively associated with symptoms or contact with psychiatry [15] [3], and others showed mixed results. For example, Nielsen et al. [53] found that boys, compared to girls, had more mental health problems in teachers’ responses, but not in self-reports. In contrast, Montgomery found that males were less likely to have symptoms of internalizing behaviors [48].

Nine studies investigated the association of socioeconomic status (SES) between health outcomes using different SES indicators, such as parental education level, being poor, starvation, self-rated SES, housing type, or disposable income. The results are inconclusive. Two of the studies reported no associations [33, 6]. One study investigated being poor and self-rated SES and found that higher SES or not being poor was related to less dissociation in the newly arrived, but the opposite was seen for the settled students. No association was found to symptoms of PTSD [23]. A Danish study by Montgomery and Foldspang [52] found that length of father’s education predicted sleep disturbances (risk increased by year; OR 1.1; 95% CI not reported) in the child. In the same study, but another paper [48], the greater duration (years) of a mother’s education in the home country was associated with reduced externalizing and internalizing behavior in the offspring. In addition, the length of the father's education (in years) increased the likelihood of being adapted [49]. Finally, the type of housing was studied in one Swedish study, but no association was found to symptoms of PTSD [58].

### Exposure to adversity

18 studies reported exposure to stressful life events or violence except the migration (Table 3). Almost all studies examined the association between exposure to some adversity and health. Most of these found a positive association between exposure to adversity and mental ill-health [2] [3] [28] [29] [27] [51] [49] [48] [76]. For example, a Danish study by Abdalla and Eklit [1] found an association between levels of exposure to violence and higher prevalence of outcomes, such as anxiety, depression, psychosomatic symptoms, nervousness, PTSD, regression, and behavioral problems, with exception of school- and eating problems and headache. Similarly, a Norwegian study by Jensen et al. [32] found that exposure to adversity was related to all internalizing symptoms, but not to externalizing symptoms. In the follow-up of the same study, a change in exposure to stressful life events also successfully predicted a symptom change in PTSD [33]. However, some other studies failed to find statistically significant associations [23] [51] [66].

**Table 3:** Review over risk and protective factors for mental and physical health, respectively, among children in the Nordic countries with a refugee background

	Authors and publication year	Title	Excluded risk and protective factors	Risk and protective factors	Health outcomes
1	Abdalla and Elklit [1]	A nationwide screening of refugee children from Kosovo		Higher age	More symptoms of PTSD,anxiety, nervousness, headache Less enuresis, eating problems
				Sex	No association
				Time since displacement	Longer time as refugee: more symptoms of depression, aggression, nervousness and psychosomatic problems Short or long time: more eating difficulties
				Exposure to violence	More symptoms of anxiety, depression, psychosomatic symptoms, nervousness, PTSD, regression and behavioral problems. For eating problems and headache the symptoms declined for those with the most exposure to violence
				Exposure to torture	More symptoms of depression, psychosomatic symptoms, regressive symptoms, PTSD, school- and conduct problems More symptoms of aggression, anxiety, nervousness and hearing problems (except for children most exposed to torture)
				Loss	More behavioral problems, anxiety, depression, aggressiveness, nervousness, PTSD, psychosomatic-symptoms, regression and PTSD. For behavioral problems the curve declines for the most affected
				Separation	More symptoms of depression, PTSD, regressive symptoms and conduct problems. More symptoms of anxiety, nervousness and aggression (except children exposed to the highest number of separations)
				Extreme poverty	More symptoms of depression, psychosomatic symptoms, regressive symptoms, PTSD, conduct problems, aggression, anxiety, nervousness and enuresis and headache
				Starvation	More symptoms of depression, PTSD, conduct problems, aggression, anxiety, nervousness
2	Almqvist and Brandell-Forsberg [2]	Refugee children in Sweden: post-traumatic stress disorder in Iranian preschool children exposed to organized violence	None	Exposure to organized violence	More symptoms of PTSD
3	Almqvist et al. [3]	Mental health and social adjustment in young refugee children 3.5 years after their arrival in Sweden	Marital discord, decreased well-being in mother, decreased well-being in father, no peer to play with, exposed to bullying	Traumatic stress exposure	More symptoms of mental ill-health
				Age	No association
				No vulnerability before exposure	More symptoms of well-being
				Parents exposure to war and persecution	Worse social adjustment
				Male sex	Worse general adaptation
				Longer time since arrival	Better social adjustment
4	Angel et al. [4]	Effects of war and organized violence on children: a study of Bosnian refugees in Sweden	Parent in need of psychiatric treatment, talking about the war, social networks	The amount of traumatic exposure	More symptoms of general anxiety, phobic and depressive symptoms, war-preoccupation (if higher traumatic exposure)
				Length of stay	No association
				Age	No association
				Sex	No association
				Being from Sarajevo	Less symptoms of behavior problems and war preoccupation (if from other city than Sarajevo)
5	Back Nielsen et al. [6]	Risk of childhood psychiatric disorders in children of refugee parents with post-traumatic stress disorder: a nationwide, register-based, cohort study	No parental psychiatric diagnosis, geographical origin of parents	Parental PTSD	More likely to have symptoms of PTSD
				Female sex	No association
				Disposable income	No association
				Geographical origin	More likely to have symptoms of PTSD if geographical origin was Middle East and central Europé, Sub-Saharan Africa or other compared to Eastern Europe and Asia
6	Berg et al. [7]	Underutilisation of psychiatric care among refugee adolescents in Stockholm	None	Residency status	More PTSD among asylum seekers than family reunification
					More use of care among asylum seekers than family reunification
				Duration in Sweden	More visits to child psychiatric services with more time in Sweden
				Income level of country of origin	More visits for high-income countries
7	Eiset et al. [14]	The health status of newly arrived asylum-seeking minors in Denmark: a nationwide register-based study	None	Sex	No association for anxiety More depression for males
				Separations	More depression and anxiety
				Loss of family member	More depression and anxiety
				Poverty and starvation	More depression and anxiety
				Exposure to violence	More depression and anxiety
8	Ekblad [15]	Psychosocial adaptation of children while housed in a Swedish refugee camp: Aftermath of the collapse of Yugoslavia	Parents who stated that they coped well with the asylum and made their own meals, apathetic or unstable mother, social support, being of pre-school age, exposure to violence, geographical origin, longer time since arrival, higher education level amongst fathers, lack of proper information about flight.^1^	Female sex	Less total symptoms of mental and somatic ill-health
				Age	More symptoms of mental and somatic ill-health with higher age
9	Gusic et al. [23]	Dissociative experiences and trauma exposure among newly arrived and settled young war refugees	None	Being poor	For newly arrived less symptoms of dissociation For settled students more symptoms of dissociation
				Accompanied	No association
				Child labor	No association
				Sex	No association
				Age	No association
				Higher socioeconomic status	For newly arrived less symptoms of dissociation For settled students more symptoms of dissociation No association to PTSD
				PTE exposure	For settled students more symptoms of PTSD For settled students more symptoms of dissociation No association for newly arrived
10	Hjern et al. [28]	Persecution and behavior: a report on refugee children from Chile	None	Persecution	More symptoms of dependency More symptoms of sleep disturbances
				Age	Dependency more common in preschool children and concentration difficulties more common in school children
11	Hjern et al. [29]	Political violence, family stress and mental health of refugee children in exile	Divorce, parent in psychiatric care, family stress, social network	Experiences of violence	More symptoms of mental ill-health
				Separations	More symptoms of mental ill-health
				Female sex	More symptoms at the first measurement but no association at second
				Age	No association
				Nationality (chilean)	No association
12	Hjern and Angel [27]	Organized violence and mental health of refugee children in exile: a 6-year follow-up	Recent family stress	Male sex	No association
				Born in Chile	No association
				Age (above 12)	No association
				Experiences of organized violence	More symptoms of mental ill-health
13	Jensen et al. [33]	Stressful life experiences and mental health problems among unaccompanied asylum-seeking children	None	Female sex	Girls scored higher on the CPSS avoidance subscale
				Region of origin (Asia vs. Africa)	No association
				Age	No association
				Time since arrival	No association
				Stressful events	More symptoms of PTSD, anxiety, depression, internalization and total symptom (HSCL total) No association to externalizing symptoms
14	Jensen et al. [33]	Development of mental health problems—a follow-up study of unaccompanied refugee minors	None	Sex	No association
				Length of stay	No association
				Length of education	No association
				Change in stressful life events	More symptoms of PTSD. No association with internalizing or externalizing problems
15	Montgomery and Foldspang [50]	Traumatic experience and sleep disturbance in refugee children from the Middle East	Fathers scolds the child more than previously	Grandparent’s violent death before the child was born	More sleep disturbances
				Mother tortured	More sleep disturbances
				Being accompanied by both parents	Less sleep disturbances
				Kurdish etnicity	More sleep disturbances
				Longer length of fathers education	More sleep disturbances
				One or both parents tortured	More sleep disturbances
16	Montgomery and Foldspang [51]	Seeking asylum in Denmark: refugee children’s mental health and exposure to violence	None	Previous refugee camp residence	More symptoms of anxiety for children without residence permit. No association for those with permit
				Having witnessed violent events	More symptoms of anxiety for children without residence permit. No association for those with permit
				Exposure to war	More symptoms of anxiety
				Having a tortured parent	More symptoms of anxiety. No association for those without permit
17	Montgomery [48]	Long-term effects of organized violence on young Middle Eastern refugees’ mental health	Having witnessed attack on others in Denmark, attending school or work, number of stressful experiences in Denmark, number of types of discriminating experiences, number of Danish friends, spelling competency, number of schools attended in Denmark	Greater duration of mother's education in the home country	Less symptoms of externalizing & internalizing behaviors
				Number of types of experiences before arrival in Denmark	More symptoms of internalizing behavior
				Higher age	Less symptoms of externalizing behavior
				Religious affiliation (both muslim and christian)	Less symptoms of internalizing behavior
				Male sex	Less symptoms of internalizing behaviour
18	Montgomery [49]	Trauma and resilience in young refugees—a 9-year follow-up study	Stressful events after the arrival, communication, attending school or work, speaks frequently to mother about problems	Number of traumatic experiences before arrival	More likely to have symptoms of mental health problems at both initial and follow-up examination (if higher number of traumatic experiences)
				Length of fathers education in the home country	More likely to not have symptoms of mental health problems at follow-up (if longer education)
19	Montgomery and Foldspang [50]	Discrimination, mental problems, and social adaption in young refugees	Parents social situation	Age	More symptoms of internalizing problems with higher age
				Nationality	No association
				Etnicity	No association
				Religion	No association
20	Nielsen et al. [53]	Mental health among children seeking asylum in Denmark—the effect of length of stay and number of relocations: cross-sectional study	Number of relocations	Longer length of stay	More mental health difficulties
				Sex	No association on the total symptom score but girls had more emotional problems and males more behavioral problems on subscale level
21	Salari et al. [58]	Using CRIES to screen for post-traumatic stress disorder in unaccompanied refugee minors	None	Type of housing in Sweden	No association
				Length of migration journey	No association
				Time spent in Sweden	No association
				Age	No association
				Country of origin (Afghanistan vs other)	No association
22	Sarkadi et al. [59]	Is the Refugee Health Screener a Useful Tool when Screening 14–18-Year-Old Refugee Adolescents for Emotional Distress?	None	Asylum status	More symptoms of emotional distress among those awaiting decision compared to those with residence permit No difference in PTSD-symptoms
				Being unaccompanied	More symptoms of distress among the unaccompanied No difference in PTSD-symptoms
23	Solberg et al. [66]	Children at risk: a nation-wide, cross-sectional study examining post-traumatic stress symptoms in refugee minors from Syria, Iraq and Afghanistan resettled in Sweden between 2014 and 2018	None	Age	No association
				Country of origin	Refugee minors from Afghanistan higher prevalence than those from Syria and Iraq
				Being unaccompanied	In the total sample unaccompanied minors has higher incidence of PTSD. When looking at different countries this was only statistically significant for Afghanistan
				Sex	No difference in PTSD-symptoms
24	Solberg et al. [65]	Health-related quality of life in refugee minors from Syria, Iraq and Afghanistan resettled in Sweden: a nation-wide, cross-sectional study	Living with parents/residential home	Age	Higher age associated with worse wellbeing
				Being unaccompanied	Unaccompanied has worse wellbeing
				Country of origin	Being from Afghanistan associated with worse well-being compared to Syria and Iraq
				Socioeconomic status	Those with good economy had better wellbeing compared to children with average or poor economy
				Sex	Girls had worse wellbeing
25	Sourander [66]	Behavior problems and traumatic events of unaccompanied refugee minors	None	Higher age	Less behavioral problems
				Sex	No association
				Nationality	No association
				Duration of flight	No association
				Persecution or death of parents	No association
26	Vervliet et al. [76]	The mental health of unaccompanied refugee minors on arrival in the host country	None	Parents still alive	No association
				Number of traumatic events	More symptoms of anxiety, depression and PTSD in males
				Age	No association

_1_Excluded because of lack of numerical data

*CPSS* child PTSD symptom scale, *CRIES* children´s revised impact of event Scale, *HSCL* Hopkins symptom checklist, *PTE* possible traumatic event, *PTSD* post-traumatic stress disorder, *vs* versus

There were also four studies investigating experiences of loss and separation*.* For example*,* a Danish study by Abdalla and Elklit [1] found an association between the number of people that the child has been separated from and symptoms of depression, PTSD, regressive symptoms, and conduct problems. In another Swedish study by Hjern, Jeppson and Angel [29] separations were associated with mental health problems at 4–6 and 17–19 months after arrival. On the contrary, there was no significant difference in mental health problems between male unaccompanied minors who lost one or both parents and those whose parents were still alive in the Verliet et al. study [76]. Similarly, a Finnish study by Sourander [66] found no association between loss and symptoms of behavior problems.

One study investigated if physiological or psychological vulnerability, or delayed development in the child before the experience of war, was associated with later changes in mental well-being [3]. The results indicated better well-being for those with no vulnerability before exposure.

### Asylum status and refugee journey

Five studies investigated if refugee status is associated with health-related outcomes with mixed results. For example, Solberg et al. [65] and Sarkadi et al. [59] found some associations, whereas in another study, being unaccompanied was not associated with PTSD and dissociation [23]. Berg et al. [7] found that children who had received residency after an asylum application had a higher incidence of PTSD compared to those who had grounds of family reunification (47.5 vs. 20.0%; *p* < 0.001). In addition, the number of health care visits among asylum seekers was higher than among those who arrived in Sweden due to family reunification.

Eight studies investigated temporal aspects of the refugee journey and resettlement. Two studies assessed the duration of the refugee journey and health outcomes with no associations [58] [66]. Findings concerning time in the country of resettlement are inconclusive. For example, a Danish study by Abdalla and Elklit [1], found that a long time as a refugee was positively correlated with depression, aggression, nervousness, and psychosomatic problems. On the contrary, Angel, Hjern and Ingelby [4] found no association between time in Sweden and total problems, generalized anxiety, or phobic and depressive symptoms. Jensen et al. [32] found no association between time in Norway and mental health problems. Similarly, the follow-up 1.9 years later [33] found that length of stay did not predict changes in symptoms of anxiety, depression, or externalizing symptoms. A study on Danish school children [53] found that children who have been asylum seekers for more than one  year have a five times higher risk of developing mental health problems measured by SDQ (OR 5.5; 95% CI 1.8–16.3).

## The family level risk or protective factors

Six studies investigated associations between parental experiences of adversity and health-related outcomes in the child with mixed results. For example, in a Swedish study by Almqvist and Broberg 1999 [3], parental exposure to adversity was associated with worse social adjustment in the child, but not with mental health problems. In a Danish study by Montgomery and Foldspang [52], mothers´ experience of torture (OR 2.4; 95% CI not reported), or one or both parent’s having experienced torture (OR 2.3; 95% CI not reported), increased the risk for sleep-disturbances in the child. Grandparents' violent death before the child was born was associated with a threefold increased risk of sleep disturbances in the child (OR 3.3; 95% CI not reported). Another paper by the same authors from the same study sample [51], found that having a tortured parent increased the risk for anxiety in children with a residence permit (OR 2.1; 95% CI 1.1–3.9) but found no significant association for those without a residence permit.

A Danish study by Back Nielsen et al. [6] investigated the association between parental PTSD and later psychiatric contact in their offspring. They found a statistically significantly increased risk among children exposed to parental PTSD of any psychiatric contact in childhood compared to refugee children without exposure to parental PTSD. There was a heightened risk among children with maternal PTSD (HR 1.62; 95% CI 1.20–2.07, *p* = 0.00), paternal PTSD (HR 1.50; 95% CI 1.20–1.90, *p* = 0.00), and PTSD in both parents with the highest risk when both parents were affected (HR 1.80; 95% CI 1.34–2.66, *p* = 0.00).

## Community and societal level risk or protective factors

11 studies assessed the role played by geographical origin, ethnicity, or nationality. Most of the studies found no significant association for geographical origin [29] [32] [50] [66] [58]. On the other hand, for example, a Swedish study by Angel, Hjern and Ingleby investigated the association between geographical origin and mental health [4]. They found that children from other cities than Sarajevo in a Bosnian sample scored higher on behavior problems and war preoccupation than children from Sarajevo. In a Danish study [6] refugee children from the Middle East and Central Europe (HR 1.42, 95% CI 1.15–1.75) and sub-Saharan Africa (HR 1.41, 95% CI 1.05–1.90) were more likely to have psychiatric contact than refugee children from Eastern Europe and Central Africa. In a Danish study by Montgomery and Foldspang, children with Kurdish ethnicity compared to other Middle Eastern ethnicities were almost twice as likely (OR 1.8, 95% CI not reported) to have sleep disturbances [52]. Also, Solberg et al. found that being from Afghanistan is associated with worse well-being compared to being from Syria and Iraq [66].

Religion was investigated in a study by Montgomery reporting that religion was associated with fewer symptoms of internalizing symptoms [48]. On the contrary, no association was found in the second paper on the same study sample  [50].

## Discussion

This is the first systematic review on resilience and risk and protective factors for mental and physical health and health-related outcomes among refugee children living in Nordic countries. In sum, the identified 26 studies on the topic included 34,080 participants from all Nordic countries except for Iceland. Most of the studies had a heterogeneous study population consisting of refugees from different countries and/or continents. Data on health outcomes were mainly collected from parents, and nearly 40% of the studies did not use standardized instruments. Six out of 26 studies reported physical health outcomes, mainly as frequencies and focusing on psychosomatic symptoms, such as headache, poor appetite, eating problems, and recurrent abdominal pain. On the other hand, all 26 studies showed data on mental health outcomes, focusing mainly on depression, anxiety, and PTSD. Notably, no study reported any data on resilience-related factors and/or outcomes. The most frequently studied risk and protective factors were age, sex, and exposure to adversity, including loss/separation.

The included studies showed associations between adversity, such as exposure to violence, experiences of war and persecution as well as losses and separations, and different mental health problems. In addition, parental mental health problems and parental exposure to adversity were identified as risk factors. Most of the studies found no association for age and sex, and mixed results were found in the rest of the studies. Other investigated risk and protective factors, such as socioeconomic status, refugee status, time in the country of resettlement, and religion, showed inconclusive findings.

The refugee children in Nordic studies included both unaccompanied and accompanied minors. In this systematic review, we used a strict definition of the target population. However, one fundamental problem in the field is the lack of stringent use of a common definition for migrant and refugee children [73]. In line with this, we noticed that in Nordic studies, first- and second-generation immigrants are often merged in analyses. Children in some excluded studies are also called refugees or migrants even though they are citizens and were born in a Nordic country. Future studies should pay more attention to clearly defining and describing the populations of interest. In addition, more nuanced analyses regarding different groups of migrants and refugees should be performed to catch the heterogeneity in detail.

Most of the studies in this systematic review used symptoms or symptom clusters such as internalizing and externalizing mental problems as outcomes instead of established diagnoses. This is in line with the methodology found in the previous [30, 37, 55] systematic reviews on refugee mental health in non-Nordic populations. In the Nordic studies, the prevalence of reported mental health problems varied considerably. For example, the prevalence of depression ranged from 2 to 45%, PTSD from 2.9 to 76.4%, and anxiety from 5 to 69%. Similar variable results have been obtained in other systematic reviews [37], whereas those with strict inclusion criteria for diagnosing mental health problems show less divergent figures [10]. In addition, differences in study populations and contexts, source of information, and chosen methods might explain the variation as suggested in previous systematic reviews focusing on violence against children in migration [34].

Notably, only two studies reported data on broader health-related outcomes such as health care utilization and health-related quality of life that is known to be associated with mental distress in refugees [74]. In addition, the lack of studies on physical health among refugee children is problematic with the known overrepresentation of various health issues [5] upon arrival to receiving countries and existing guidelines with a recommendation for  targeted health assessments. However, several reported findings related to health outcomes in the Nordic studies seem to align with typical development in children. For example, most children grow out of enuresis over time or become less dependent on their parents. Since there is no comparison to the expected development of children in these studies, it is uncertain if the findings reflect a risk for refugee children—or just normal development in children. In addition, it remains unclear whether refugee children differ from children in general or if the findings simply reflect known sex differences in children’s and adolescents´ mental health [20, 68, 75, 78]. This also reflects a general problem in the reviewed studies: a lack of theoretical reasoning explaining how and why the research questions are relevant for the population of migrant and refugee children. For example, when the research question concerns associations already established among children in general, the reason why it’s relevant to study these questions among migrant children and a rationale for expected deviations from the normative development should be stated. In addition, more stringent, and nuanced reporting and discussion on effects in terms of strengths, and practical or clinical significance, rather than only statistical significance is warranted.

The Nordic studies have investigated many different risk and protective factors for health. The studied factors, for example, age, sex, exposure to adversity/violence, time since displacement as well as parental mental health and education, are similar to those studied in non-Nordic populations [55, 60] including displaced and refugee children resettled in low-income and middle-income countries. The reported risk and protective factors were mostly on an individual level, age and sex are among the most studied factors. A recent systematic review on gender differences in mental health among unaccompanied refugee minors indicates that the female gender is associated with higher vulnerability towards certain mental health problems, such as depression  [47]. This data suggests that a closer analysis of age-related gender differences in various mental health outcomes among refugee children residing in the Nordic region may be warranted.

Along with age and sex, exposure to adversity was investigated in many Nordic studies. However, the definition of adversity differed between the studies. Most frequently, adversity was defined as exposure to violence and/or war experience, and several types of exposures are reported (Table 2). However, in some of the included studies, being a refugee or asylum seeker is itself considered adversity. However, this assumption is not scientifically tested nor discussed in any article. Thus, the relative contribution of being a refugee on top of the adverse events that some children experience is unclear. Should the migrant status be considered as exposure, a risk factor, or does it moderate or mediate different outcomes? Could it be a protective factor in some circumstances? We agree with other researchers who have emphasized a need for larger conceptual clarity regarding adversity in general [34] and adversities connected to being in migration [44, 45] specifically.

In this systematic review, we applied a strict definition of risk and protective factors. We required an association between an exposure and outcome, that risk and protective factor occurred before the outcome, and that there was a clear reason why a factor should be considered as a risk or protective factor. Due to the strict definition, many putative community and social level risk and protective factors such as social support and school attendance were excluded. None of the included community or social risk and protector factors was specifically connected to the Nordic welfare setting. Identifying risk and protection factors on a societal level is important to tailor interventions in society to promote health among refugee children, as recently pointed out in a systematic review by Höhne et al. [31].

No studies on resilience were identified, even if the concept is well-known, widely used, and to some extent covered in systematic reviews on refugees [40]. One of the explaining factors might be the lack of a clear definition of the concept of resilience. In recent years, resilience research has moved from a focus on the individual child to processes and environments [72]. This is not reflected in the reviewed articles, where the focus mainly is on the individual child. There is also a lack of longitudinal studies that could address the temporal aspects of resilience and the interplay of different factors over time [13, 35]. One could assume that arriving in a host country with the social benefits that Nordic countries have for children, such as subsidized child-care and free health care, should foster resilience, compared to other countries without a welfare system. It is still not clear how much the societal system contributes to resilience. Besides the welfare system, many other societal factors could contribute to refugee children's health and ill-health, for example, the amount of racism in the general population, temporary resident permits, and segregation both in schools and in residential areas. These factors are not necessarily better in Nordic countries compared to other western countries. Further studies on resilience and its promotion are warranted.

### Strengths and limitations

The main strength of this study is the synthesis of data from Nordic studies on health outcomes, resilience, as well as risk and protective factors. Adherence to PRISMA standards [46] allows rigorous methodology, complemented with AMSTAR analysis of reliability, validity, and quality of the report [62]. Another strength is the use of a clear definition of risk and protective factors. Defining these factors is currently not standard and might lead to confusion, since many of the proposed risk and protective factors could be outcomes.

Nevertheless, this study has several limitations. The analysis focuses only on studies conducted in high-income Nordic countries, which limits the generalizability to low- and middle-income settings. The cross-sectional study design used in several studies is a major limitation in the identification of risk and protective factors that could have been relevant for outcomes over time. In addition, the causality of the predictive factors identified in this type of study should be taken with caution. Longitudinal follow-up studies might have been more informative but due to the restriction of the analysis to individuals 18 years or younger, some studies that followed children into adulthood were excluded.

The included studies vary considerably in design, samples sizes, and origin of the participating refugee children. In addition, the use of parental reports as the main data source, non-validated instruments, and heterogeneity of reported outcomes limit conclusions that can be drawn from this study. The limitations in design and statistical methods might be explained by the fact that most of the studies are old. The data did not allow any meta-analysis, because the study designs and data were very diverse. In addition, the results were not always presented very precisely. The lack of meta-analysis limits the ability to draw definitive conclusions. Several well-powered studies using clear definitions, same or similar data collection instruments, and outcomes are required for future meta-analysis [25].

A more detailed reporting on risk and protective factors on the level of association between a factor and outcome, a risk that occurred before the outcome, and a stated logical reason for a factor to be considered as a risk or protective factor would have increased the quality of many studies. To compensate for limits in reporting, a secondary assessment of factors was done based on the mentioned criteria. This resulted in the exclusion of several reported factors (Table 3) that might still play a role in health outcomes. Yet another limitation is the predominant focus on risk factors and negative health outcomes. No studies on resilience were found and reports of non-ill-health were missing. These aspects should be paid more attention to in future studies.

## Conclusions

We conclude by identifying gaps not only in terms of risk and protective factors but also with an inconsistency in the research field regarding common terms, such as definitions of adversity, migrant and refugee populations, mental health outcomes, and resilience. To sum up, this review highlights the existing data from Nordic studies on health outcomes and risk and protective factors in migrant children with a refugee background. Due to a lack of consistency in key definitions and great heterogeneity in the included studies, it is hard to draw any conclusions about if and how the Nordic welfare model contributes to refugee children’s health and resilience. Special attention should be paid to defining golden standards and common definitions to further improve the quality of the published studies on the topic.

### Supplementary Information

Below is the link to the electronic supplementary material.

Supplementary file1 (DOCX 17 KB)

## Data Availability

All relevant data is available upon request.
