# Supplementary material for: A systematic review of studies on resilience and risk and protective factors for health among refugee children in Nordic countries
Source: Eur Child Adolesc Psychiatry. 2022 Apr 20;33(3):667–700. doi: 10.1007/s00787-022-01975-y (PMC10894096; doi:10.1007/s00787-022-01975-y)
Supplement: Supplementary file 1 — Supplementary file1 (DOCX 17 KB) [file 787_2022_1975_MOESM1_ESM.docx]

**Supplementary information**

**Appendix 1.** Search strategy

((((((((((((((development[Title/Abstract]) OR protective factor[Title/Abstract]) OR adaptation[Title/Abstract]) OR modifying factor[Title/Abstract]) OR vulnerability factor[Title/Abstract]) OR risk factor[Title/Abstract]) OR recovery[Title/Abstract]) OR adjustment[Title/Abstract]) OR resilience[Title/Abstract]) OR health[Title/Abstract]))

OR ((((((((((((psychological resilience[MeSH Terms]) OR child development[MeSH Terms]) OR protective factors[MeSH Terms]) OR adaptation, psychological[MeSH Terms]) OR risk management[MeSH Terms]) OR mental health recovery[MeSH Terms]) OR social adjustment[MeSH Terms]) OR emotional adjustment[MeSH Terms]) OR risk adjustment[MeSH Terms]) OR Sense of Coherence[MeSH Terms]) OR post-traumatic growth[MeSH Terms]) OR self-management[MeSH Terms]))

**AND**

((((((sweden[Affiliation] OR norway[Affiliation] OR finland[Affiliation] OR denmark[Affiliation] OR iceland [Affiliation]

OR ((swedish[Language] OR norwegian[Language] OR danish[Language] OR finnish[Language] OR icelandic[Language])))

OR

(((Scandinavian and Nordic Countries[MeSH] OR sweden[MeSH] OR norway[MeSH] OR denmark[MeSH] OR finland[MeSH] OR iceland[MeSH])))

OR

((sweden[Title/Abstract] OR swedish[Title/Abstract] OR norway[Title/Abstract] OR norwegian[Title/Abstract] OR denmark[Title/Abstract] OR danish[Title/Abstract] OR finland[Title/Abstract] OR finnish[Title/Abstract] OR iceland[Title/Abstract] OR icelandic[Title/Abstract] OR nordic[Title/Abstract] OR scandinavia[Title/Abstract] OR scandinavian[Title/Abstract])))))

**AND**

(((((infant[MeSH] OR child[MeSH] OR adolescent[MeSH])))

OR

((pediatric[Title/Abstract] OR pediatrics[Title/Abstract] OR paediatric[Title/Abstract] OR paediatrics[Title/Abstract] OR child[Title/Abstract] OR children[Title/Abstract] OR childs[Title/Abstract] OR childhood[Title/Abstract] OR daughter[Title/Abstract] OR daughters[Title/Abstract] OR son[Title/Abstract] OR sons[Title/Abstract] OR boy[Title/Abstract] OR boys[Title/Abstract] OR girl[Title/Abstract] OR girls[Title/Abstract] OR boyhood[Title/Abstract] OR girlhood[Title/Abstract] OR teenager[Title/Abstract] OR teenagers[Title/Abstract] OR teen[Title/Abstract] OR teens[Title/Abstract] OR adolescence[Title/Abstract] OR adolescent[Title/Abstract] OR adolescents[Title/Abstract] OR youth[Title/Abstract] OR youths[Title/Abstract] OR minor[Title/Abstract] OR minors[Title/Abstract]))))))

**AND**

(((((“emigrants and immigrants”[MeSH] OR "transients and migrants"[MeSH] OR refugees[MeSH])))

OR

((migrant[Title/Abstract] OR migrants[Title/Abstract] OR immigrant[Title/Abstract] OR immigrants[Title/Abstract] OR "newly arrived"[Title/Abstract] OR "displaced person"[Title/Abstract] OR "displaced persons"[Title/Abstract] OR "asylum seeker"[Title/Abstract] OR "asylum seekers"[Title/Abstract] OR refugee[Title/Abstract] OR refugees[Title/Abstract]))))
